# Biomedical event extraction with a novel combination strategy based on hybrid deep neural networks

**DOI:** 10.1186/s12859-020-3376-2

**Published:** 2020-02-06

**Authors:** Lvxing Zhu, Haoran Zheng

**Affiliations:** 1 School of Computer Science and Technology, University of Science and Technology of China, Huangshan Road, Hefei, 230026 People’s Republic of China; 2 Anhui Key Laboratory of Software Engineering in Computing and Communication, University of Science and Technology of China, Huangshan Road, Hefei, 230026 People’s Republic of China; 3 Anhui Province Key Lab. of Big Data Analysis and Application, University of Science and Technology of China, Huangshan Road, Hefei, 230026 People’s Republic of China

**Keywords:** Event extraction, Biomedical text, Deep learning Neural network

## Abstract

**Background:**

Biomedical event extraction is a fundamental and in-demand technology that has attracted substantial interest from many researchers. Previous works have heavily relied on manual designed features and external NLP packages in which the feature engineering is large and complex. Additionally, most of the existing works use the pipeline process that breaks down a task into simple sub-tasks but ignores the interaction between them. To overcome these limitations, we propose a novel event combination strategy based on hybrid deep neural networks to settle the task in a joint end-to-end manner.

**Results:**

We adapted our method to several annotated corpora of biomedical event extraction tasks. Our method achieved state-of-the-art performance with noticeable overall F1 score improvement compared to that of existing methods for all of these corpora.

**Conclusions:**

The experimental results demonstrated that our method is effective for biomedical event extraction. The combination strategy can reconstruct complex events from the output of deep neural networks, while the deep neural networks effectively capture the feature representation from the raw text. The biomedical event extraction implementation is available online at http://www.predictor.xin/event_extraction.

## Background

PubMed recorded over 28 million papers in 2018 [1] which reflects the rapid growth of the biomedical literature. The knowledge and discoveries reported in the biomedical literature receive substantial attention, but the large volume of the literature poses a challenge to information retrieval; therefore, text mining has become an in-demand technology and a popular research focus. Event extraction, which is an effective way to represent the structured knowledge from unstructured text [2], is a fundamental technology for text mining. However, event extraction is particularly difficult due to the complex and arbitrary structure of events in biomedicine, so related research is urgently needed [3].

The definition of a biomedical event, according to the BioNLP [4], consists of (1) a trigger word that indicates the existence of an event and belongs to a certain event type and (2) multiple arguments in which an argument can be viewed as a relation between the event triggers and entities or other event, and each argument has an argument type as well. Therefore, the task of event extraction is to recognize the event triggers with their arguments from the raw text.

**Fig. 1:**
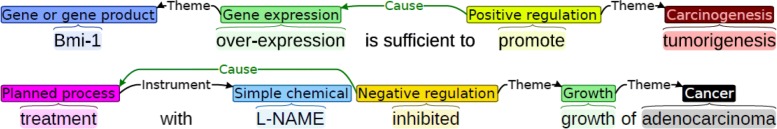
A sentence with visualized events, provided by BioNLP-ST2013

We illustrate biomedical events with Fig. 1 as example. The word “promote” is an event trigger of the event type *Positive Regulation*. This event has a *Theme* argument linked to the word “tumorigenesis”, which is an entity of *Carcinogenesis* type, and an *Cause* argument linked to “over-expression”. Notice that some events can be the argument for other events, i.e., a nested structure, such as “over-expression” serving as an *Gene Expression* event trigger as well as an argument of *Positive Regulation* event. Therefore, the event can be viewed as a directed graph in the text for which the node of graph is the event trigger or entity and the directed edges indicate the arguments.

Since biomedical event extraction was defined as a standard task, various methods have been proposed. Most previous work can be classified into three types: rule-based approaches [5, 6], traditional shallow machine learning models and deep learning models. The Turku Event Extraction System (TEES) [7, 8] is a biomedical event extraction system that uses rich features from dependency parsing. The TEES utilizes a step-wise approach based on multi-class SVMs by breaking down the whole task into straightforward consecutive graph node and edge classification tasks. The EventMine [9] is a similar SVM-based pipeline method with handcrafted features. Majumder et al. [10] exploited a stacking model for biomedical event extraction. The system uses SVC, SGD and LR as the base-level classifiers and takes SVC as the meta-level classifier. A transition-based model for event extraction [11] is another approach leveraging a structured perception for encoding and decoding with a beam search to find the global best prediction.

In recent years, deep learning methods have been applied to this task that extend the feature representation from text and promote the performance. Wang et al. [12] proposed a convolutional neural network (CNN) with multiple distributed features for biomedical event extraction. The distributed features contain not only the word embedding but also trigger types, POS labels and topic representation. Li et al. [13] utilized dependency-based word embedding and a parallel multi-pooling convolutional neural network to extract biomedical events. This approach reserves more information by pooling the multi-segment of a sentence divided by word triggers and arguments. Björne and Salakoski [14] integrated CNN into the original TEES to supply more features, and replaced the SVM classifier with dense layers, which suggested that the inclusion of the neural network significantly enhanced the performance. Li et al. [15] proposed a framework that using gated recurrent unit networks with attention mechanism to extract biotope and bacteria events.

However, deep learning methods are still rarely used for biomedical event extraction, which is partially due to the complexity of the task-specific event structures. More deep models have focused on the sub-tasks of event extraction such as event trigger detection [16–18] and relation classification [19–22], and most of these models obtained superior performance compared to traditional shallow methods.

Despite the success of existing methods in biomedical event extraction, they generally suffer from two limitations. First, most of them heavily rely on manually designed features and usually need complicated natural language processing (NLP) from external NLP toolkits with poor generalizability. Second, these methods organize the task in a pipeline manner and separate it into independent sub-tasks, which simplifies the problem but ignores the interaction between the sub-tasks and makes the process prone to accumulating errors.

Due to the aforementioned limitations, we propose a biomedical event extraction method with a novel combination strategy based on deep neural networks. Our method detects the candidate event triggers and relations from raw text with recurrent neural networks (RNN) and convolutional networks (CNN), and then the Combination Strategy (CS) constructs the event from the detected results by solving an optimization problem. The proposed method takes advantage of neural networks that can represent features from word embedding in semantic space [23] and removes the reliance on feature engineering. The CS, which integrates global information to optimize a penalty, alleviates the error accumulation.

We evaluated our method with three common biomedical event extraction tasks: the Multi-Level Event Extraction (MLEE) [24], Cancer Genetics (CG) and Pathway Curation (PC) from BioNLP Shared Task 2013 (BioNLP-ST2013) [4]. Our method outperforms the state-of-the-art methods for all of these tasks according to overall F1 scores. The experimental results demonstrate the effectiveness and generalizability of the hybrid networks and the CS. Additionalty, our method only needs a minimized task-specific configurations without the adjustment on method, which makes it easy to facilitate for various biomedical event extraction tasks.

The contributions of this paper are summarized below:

- Describes the first attempt to use hybrid deep neural networks (CNN and RNN) aimed at achieving biomedical event extraction.
- Proposes a novel combination strategy to integrate the detected triggers and relations in an optimized manner.
- Utilizes end-to-end learning as well as avoids reliance on manual feature design and external NLP packages.

## Results

### Dataset

We trained and evaluated our method using three common annotated datasets: CG, PC and MLEE. Each dataset is initially divided into three parts: training, development and test sets, and the statistics of these datasets are listed in Table 1. CG data concerns the extraction of events relevant to cancer, including molecular foundations, cellular tissue, and organ-level effects. PC data targets reactions relevant to the development of biomolecular pathway models. MLEE data focuses on events across multiple levels of biological organization from the molecular level to the organ system level. All of these datasets have provided entity labels for each word so that task can focus on targeting event extraction.

**Table 1:** Statistics of datasets

Dataset	Entity type	Entity	Event type	Event	Word	Document (training)	Document (development)	Document (test)
CG	18	21683	40	17248	129878	300	100	200
PC	4	15901	23	12125	108356	260	90	175
MLEE	14	8291	28	6677	56588	131	44	87

The pre-processing is simple; we only split each document into sentences and tokenized them into sequences of words, which does not rely on any additional NLP toolkits.

### Training

The main hyper-parameters, which were tuned on the development set, were set as follows: learning rate = 0.007, ratio of class weight of positive and negative classes for TR and RC was 5:1, weight decay = 0.0002, batch size = 16, *α* = 0.5, *β* = 0.25, *γ* = 0.125, *threshold*_*t*_=−2.0 and *threshold*_*r*_=−2.0. The other hyper-parameters of our model are listed in Additional file 1: Table S11. We set *k*=10 in inverse sigmoid decay function. The activation function was leaky-relu. The optimizer we used was Adam. We used 5 single models for ensemble learning. Some tricks were employed, including using pretrained Char-level CNN and word embedding on large external corpus [25], Xavier initialization for neural layers [26], dropout in LSTM and undersampling in EE. The undersampling is to keep the class balance of positive/negative event samples in each sentence.

**Fig. 2:**
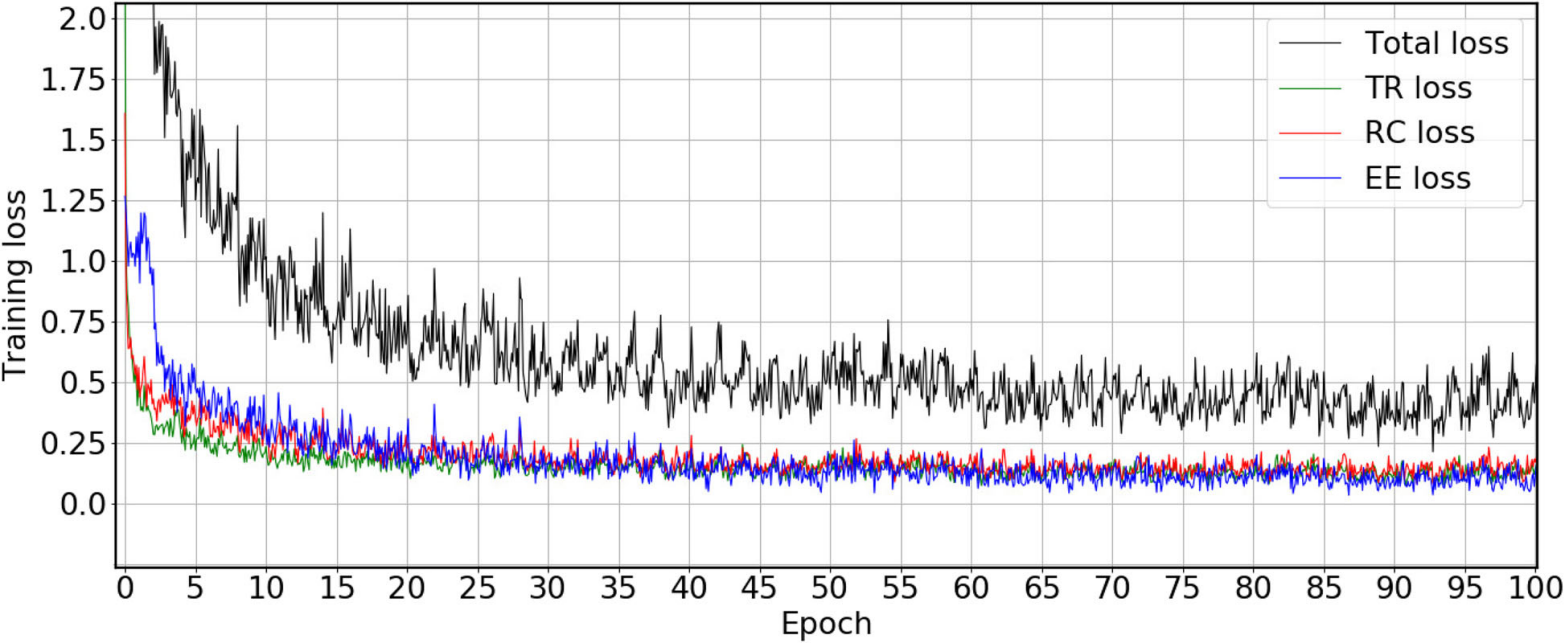
The sequence of training loss within 100 epochs

The final model were trained by the union of training and development set through 100 epochs for each task and the loss curve on CG corpus is shown as Fig. 2. The loss of each module was calculated individually and the gradient of them was propagated simultaneously to update parameters at the end of every batch. The loss of TR declines first, followded by the loss of RC, while the loss of EE has the slowest change. This phenomenon is reasonable because the latter two losses rely on the former detected results. The black curve is the total loss that sums up of loss from TR, RC and EE. The oscillation of the loss curve is due to the variance in the length of sentences and different number of events among the batches. The figure shows that the model has converged after 100 training epochs. The 100-epochs training on CG corpus (400 documents) takes about 12 h on an i7-7700 CPU. The prediction of a single model for each document takes about 20 s on average and ratio of time consuming of each module (TR, RC, EE and CS) is 55.97%, 20.75%, 23.06% and 0.23%, respectively.

### Performance

We used standard recall, precision and F1 scores as evaluation metrics. The event was regarded as true-positive only if both trigger and arguments were detected correctly. Our evaluation followed the primary criteria, i.e., approximate span matching and approximate recursive matching [27].

Table 2 shows the overall performance of our method and other state-of-the-art methods for CG, PC and MLEE on test set. RelAgent [6] is a linguistically motivated rule-based system to extract biomedical events. NCBI [5] uses an approximate sub-graph matching-based approach. Zhou and Zhong [28] utilized a semi-supervised learning framework with un-annotated corpora. The TEES [7, 8] and EventMine [9] are both SVM-based pipeline models with hand-designed rich features. The TEES CNN [14] is the upgraded version of TEES coupled with CNN and uses mixed 5 model ensemble with randomized train/development set split. Wang et al. [12] and Li et al. [13] both developed convolutional network-based methods.

**Table 2:** Comparison of overall performance on CG, PC and MLEE task (test set)

	CG			PC			MLEE		
Methods	Recall	Precision	F1 Scores	Recall	Precision	F1 Scores	Recall	Precision	F1 Scores
RelAgent [6]	41.73	49.58	45.32	-	-	-	-	-	-
NCBI [5]	38.28	58.84	46.38	-	-	-	-	-	-
Zhou and Zhong [28]	-	-	-	-	-	-	59.19	55.76	57.41
TEES [8]	48.76	64.17	55.41	47.15	55.78	51.10	-	-	-
EventMine [9]	48.83	55.82	52.09	52.23	53.48	52.84	49.56	62.28	55.20
Wang et al. [12]	-	-	-	-	-	-	56.23	60.65	58.31
Li et al. [13]	-	-	-	-	-	-	53.61	67.23	59.65
TEES CNN [14]	50.77	66.55	57.60	50.34	62.16	55.62	-	-	-
Proposed	51.91	65.81	58.04	50.65	61.95	55.73	55.02	66.08	60.05

Table 2 shows that our method achieved the highest F1 scores for all three datasets by 58.04% for CG, 55.73% for PC and 60.05% for MLEE respectively, which suggests the effectiveness and generalization ability of the hybrid networks and CS. We conducted *student’s t-test* [29] on the best existing F1 score and F1 scores of our proposed method in multiple runs. The results indicate that the improvements are statistically significant on CG and MLEE task with *p*-value <10^−3^, and *p*-value on PC task is 0.062 (the detailed statistics are listed in Additional file 1: Table S7–S9). The precision was dramatically higher than the recall in all datasets, which was probably due to the highly diverse event schemes and insufficient training set.

Table 3 shows the detailed performance for our method and other existing methods for grouped event categories of CG. As shown in Table 3, our method outperformed other methods for all the event categories. The most significant improvement was for *Modification* events, and these events relied highly on the global contextual information that was modelled precisely by the recurrent network of EE. Through vertical comparison, the lower scores for *Regulation*, *Planned Pro* and *Modification* compared to those of other categories were due to their nested structure, i.e., these events usually took other events as arguments, which were more difficult to correctly detect. The full detailed performance for CG, PC and MLEE is listed in Additional file 1: Table S4–S6.

**Table 3:** Detailed performance comparison on CG

Event Class	TEES	EventMine	NCBI	RelAgent	Proposed
Anatomical	77.20	71.31	73.68	70.82	79.78
Pathological	67.51	59.78	54.19	48.14	68.46
Molecular	72.60	72.77	67.33	60.72	73.16
General	52.20	53.08	44.70	40.89	59.45
Regulation	43.08	39.79	29.21	35.58	44.31
Planned Pro	39.43	40.51	34.28	28.57	48.15
Modification	34.66	29.95	0.00	30.88	39.67

## Discussion

### Alternatives comparison

Before the proposed method was determined, we conducted ablation experiments on several variations of the proposed method to validate the effectiveness of each part of the method. Table 4 shows results of the ablation study, which evaluated the ensemble learning, the EE module, CS algorithm, threshold setting and Char-CNN module. In these experiments, methods were trained on training set and tested on development set. The Single-model is the proposed method in singleton without ensemble learning. The Single-pipeline-model seperates TR, RC and EE into independent networks without parameter sharing. The Combination-rule-single does not use the EE module and replaces the CS with a rule-based method to assign only one event to each detected event trigger. The Combination-rule-all similarly replaces the CS with another rule-based method to generate all possible combinations from triggers and relations. The EE-probability directly uses the probability outputing from TR, RC and EE to determine the final events instead of using CS, i.e., the method extracts events simply by the classification results of each network modules (setting all thresholds to 0). The Zero-threshold resets both *threshold*_*t*_ and *threshold*_*r*_ to zero and keeps other setting same as proposed method. The Without-CharCNN removes the Char-CNN module from the method. The Single-model-pipeline and Without-CharCNN need retrain the neural networks while other methods do not retrain the networks since they only change the settings in post-processing steps.

**Table 4:** Performance comparison across variations of our method on development set

	CG			PC			MLEE		
Methods	Recall	Precision	F1 Scores	Recall	Precision	F1 Scores	Recall	Precision	F1 Scores
Proposed	51.29	63.34	56.68	49.32	59.90	54.10	53.53	62.34	57.60
Single-model	48.34	55.73	51.77	48.43	52.32	50.30	49.96	55.64	52.65
Single-pipeline-model	47.68	54.80	51.00	47.49	52.95	50.07	48.43	53.73	50.94
Combination-rule-single	50.50	57.46	53.75	46.92	55.94	51.04	48.09	55.28	51.43
Combination-rule-all	54.51	52.05	53.25	50.21	47.69	48.92	52.51	48.77	50.57
EE-probability	49.16	64.22	55.69	46.74	60.60	52.77	48.43	63.72	55.03
Zero-threshold	50.74	62.24	55.90	48.90	57.06	52.66	53.02	62.30	57.29
Without-CharCNN	50.57	62.18	55.78	46.08	55.08	50.18	51.57	63.52	56.93

As shown in Table 4, the proposed method achieved higher F1 scores than other alternative variations. The contrast results of Single-model show that the ensemble learning noticeably improved the F1 performance by 3%-5%, which was mainly contributed by the improvement in precision of 6%-8%. Since the randomness in the training led to variance among the different runs (which also caused performance differences by several percentage points, e.g., the F1 scores by multiple runs of Single-model on CG have a standard deviation of 0.532%), the ensemble could eliminate the variance and obtain higher precision. The F1 scores of Single-model-pipeline sightly declined by 0.2%-1.6% that suggests parameter sharing is effective and efficient (the training of Single-model-pipeline takes longer time than proposed joint model). The performance of Combination-rule-single decreased markedly by 3%-6% compared to the proposed method, especially for recall because the method only assigned one event for each detected trigger and multiple events associated with one trigger were discarded through its rule (such multiple events accounts for 59.7% of total events in CG corpus, for example). The Combination-rule-all obtained high recall that is even higher than the recall of our proposed method on CG and PC tasks, but it suffered from much lower precision and the F1 scores decreased markedly because it constructed too many incorrect events. Both of these ablation tests show that the EE module is valuable. Removing EE module yeilded a 0.8%-2.6% decline of F1 score by EE-probability, which indicates that the CS contributes a positive effect by optimizing the penalty value. However, EE-probability obtained higher precision because the method applied stricter extraction. EE-probability assigned an event only when all of TR, RC and EE modules returned positive classification results, thus the detected events were lesser then the proposed method did (e.g., 2225 vs. 2354 on CG development set). Additionally, the comparison with the Zero-threshold demonstrates that setting a lower threshold for the candidate triggers and relations can construct more valid events and promote overall performance. The contrast results in the last row demonstrate that the Char-CNN module is beneficial to the overall performance by providing the lexical features.

### Error and limitation analysis

We divided extraction errors into five types, including wrong trigger label, wrong trigger span, wrong arguments, redundant arguments and other errors. Detailed statistics for the errors in prediction phase are listed in Table 5. The statistics suggested that the most common error type was the wrong trigger span, which constituted about half of the total errors, indicating that the range of trigger words is the most difficult information to detect.

**Table 5:** Statistics for the extraction errors in CG/PC/MLEE

Corpus	Wrong T_Label	Wrong T_Span	Wrong Argu	Redundant Argu	Other Error	Total Error
CG	5.09%	12.11%	9.57%	5.65%	3.25%	35.67%
PC	4.75%	18.28%	4.47%	5.72%	4.22%	37.44%
MLEE	2.38%	15.72%	5.48%	6.38%	3.97%	33.93%

* The statistics are derived by training method on training set and testing on development set of CG/PC/MLEE.

* The *Wrong T_Label* represents the event triggers with the wrong assigned label. The *Wrong T_Span* represents the range of the trigger words that were wrong (including detected triggers that do not exist in the gold standard). The *Wrong Argu* indicates that the event trigger was correctly detected but the arguments were wrongly assigned. Similarly, the *Redundant Argu* indicates that redundant arguments were assigned for correctly detected triggers.

Moreover, similar to other works, two special cases were ignored to simplify in our method. First, a few events had trigger words and arguments spanning across more than one sentence, but our method only detects events within a single sentence. Secondly, a few words were associated with more than one event trigger labels, but our method could assign only one trigger label to them. Ignoring such cases could cause a performance reduction, but these cases are relatively rare (approximately 2% - 4.5%) and had limited effect (see Additional file 1: Table S10).

Some limitations still exist and need further improvement. Our method is based on a deep neural network with a large number of learnable parameters but the training set with hundreds of documents is somewhat insufficient. The tuning of hyper parameters relied on grid searching in a development set, which was time consuming.

## Conclusions

In this paper, we present deep neural networks coupled with a combination strategy to extract biomedical events. Our method detects the event trigger and classifies the relations jointly while taking advantage of deep neural networks that extract feature representation automatically and do not rely on manual feature engineering. This novel Combination Strategy integrates the outputs from different stages to construct the events in an optimization manner, which alleviates the error accumulation. The evaluation results show that our method has achieved state-of-the-art performance compared to existing methods, which indicates that the Combination Strategy and the deep neural networks in our method are effective. In the future, we plan to extend our method with semi-supervised learning to address the insufficiency of the training corpora. Since biomedical text mining is a desirable technology for converting the large number of articles to structured information at high-layer semantics, we believe the proposed method has the potential to facilitate event extraction in broad, real-world scenarios for researchers.

## Methods

**Fig. 3:**
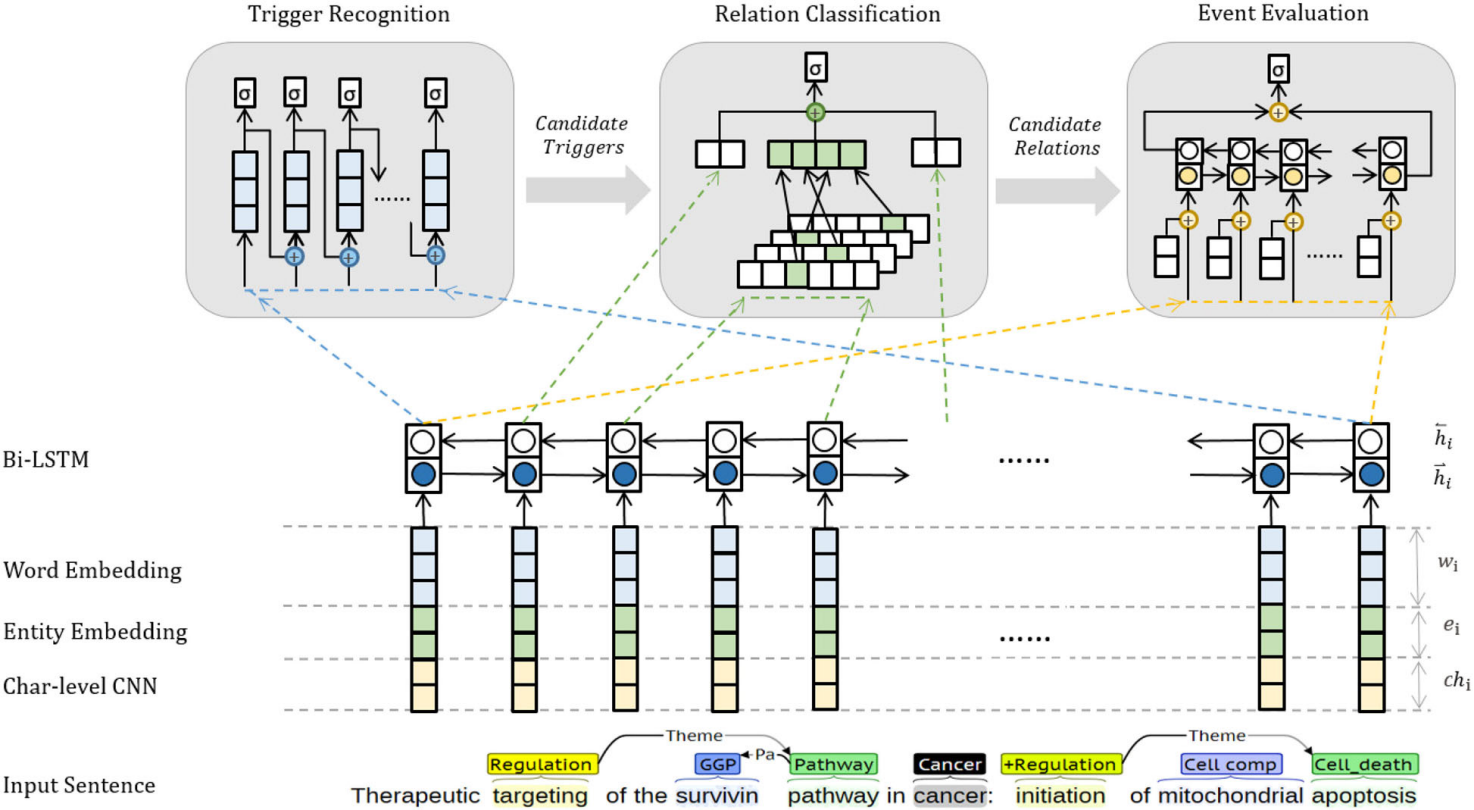
The overall networks structure (CharCNN, BiLSTM, TR, RC, EE), some components are omitted for brevity(detailed structures are shown in Additional file 1: Figure S1–S3)

We apply end-to-end supervised deep learning for event extraction. The overall architecture of the networks is illustrated in Fig. 3, which consists of 5 modules: the Character-level CNN (CharCNN), Bi-directional LSTM (BiLSTM), Trigger Recognition (TR), Relation Classification (RC) and Event Evaluation (EE). The CharCNN and BiLSTM encode a sentence into a sequence of feature vectors. The TR, RC and EE are stacked on BiLSTM and determine the type and probability of each event trigger, relation and candidate event, respectively. These modules are trained simultaneously in a joint manner, which can benefit from parameter sharing [19, 21]. Finally, the outputs of these modules are integrated into the CS, which is a post-processing step that is applied in prediction phase to generate the final events.

We cast the argument assignment as a relation classification task, so we use the term relations instead of arguments in the rest of this paper.

### Character-level cNN

Character-level CNN (CharCNN) extracts the character-level features of each word. The module is inspired by previous work [21] that has been shown to be effective due to the ability to capture the morphological information [30].

For each word, the module first looks up an embedding layer to get the vector representation of each character. Let the sequence of embedding vectors be $V^{(c)} = \left \{v^{(c)}_{1,}v^{(c)}_{2},...,v^{(c)}_{n}\right \}$ where $v^{(c)}_{i}$ is the vector of *i*-th character. Then, the sequence is fed to convolution layer, which is computed by:

1 $$ y^{(c)}_{i} =f\left(conv\left(W_{1},V^{(c)}_{i:i+k}\right)+b_{1}\right),   $$

where *k* denotes the kernel size, *conv*(·,·) is the convolutional operator, *f*(·) is the activation function, $W_{1}\in \mathbb {R}^{d\times k\times nc}$ is the parameter of the convolution layer where *nc* is the number of output channels and $b_{1}\in \mathbb {R}^{nc}$ is the bias vector. Therefore, we obtain the representation matrix $y^{(c)}\in \mathbb {R}^{n\times nc}$ for an *n*-length word. To obtain the fixed length representation of the word, the adaptive max pooling is then applied to the output vector:

2 $$ ch_{j} = \max_{1\leq i\leq n}y^{(c)}_{ij},   $$

where $ch\in \mathbb {R}^{nc}$ is the char-level representation of the word.

### Bi-directional lSTM

The Bi-directional LSTM (BiLSTM) encodes a sentence into a list of hidden vectors. The LSTM can model the long-distance dependency that benefits from its memory and forget blocks, and a signal from two directions helps the module sense the context [31].

Given a sentence with *n* words, the word embedding layer maps each word into a vector as *w*_*i*_. Similarly, the entity label of each word is also mapped to vector *e*_*i*_ by the entity label embedding layer. We have obtained the character-level representation of each word denoted as *ch*_*i*_ from Char-CNN. Then, the above vectors are concatenated and denoted as *v*_*i*_=[*w*_*i*_,*e*_*i*_,*ch*_*i*_]. The vector representation of *n* words forms *V*={*v*_1_,*v*_2_,...,*v*_*n*_}, and then it is fed to the LSTM layers with two parallel (forward and backward) directions. The computation of the LSTM layer at the time step *i* is:

3 $$ \begin{aligned} & i_{i} = \sigma (W_{jj}v_{i}+b_{jj}+W_{hj}h_{i-1}+b_{hj}), \\ & f_{i} = \sigma (W_{jf}v_{i}+b_{jf}+W_{hf}h_{i-1}+b_{hf}), \\ & g_{i} = \tanh(W_{jg}v_{i}+b_{jg}+W_{hg}h_{i-1}+b_{hg}), \\ & o_{i} = \sigma (W_{jo}v_{i}+b_{io}+W_{ho}h_{i-1}+b_{ho}), \\ & c_{i} = f_{i}\cdot c_{i-1}+i_{i}\cdot g_{i}, \\ & h_{i} = o_{i}\cdot \tanh(c_{i}),  \end{aligned}  $$

where *i*_*i*_,*f*_*i*_,*g*_*i*_,*o*_*i*_ and *c*_*i*_ are the input gate, forget gate, intermediate state, output gate and cell state, respectively. *σ*(·) is the sigmoid function. $h_{i} = \left [\overrightarrow {h_{i}},\overleftarrow {h_{i}}\right ]$ is the hidden vector from LSTM at time step *i*, which consists of two directions. Finally, we obtain the sentence encoding sequence $H = \{h_{1},h_{2},...,h_{n}\}\in \mathbb {R}^{2hd\times n}$ where *hd* is the hidden size. Additionally, we also obtain the sequence of entity label embedding *E*={*e*_1_,*e*_2_,...,*e*_*n*_}.

### Trigger recognition

We cast the Trigger Recognition (TR) as a sequence labelling task. The TR module receives the output of BiLSTM and assigns an event label to each word in the sequence in the BILOU scheme [32].

Given the input sequence *H*={*h*_1_,*h*_2_,...,*h*_*n*_}, we assign the label in a greedy manner from left to right. At the time step *i*, we concatenate the encoded vector *h*_*i*_ and previous event trigger label vector *t*_*i*−1_ into *x*_*i*_=[*h*_*i*_,*t*_*i*−1_] and then send them into a linear layer and a log softmax layer, which is written as:

4 $$ \begin{aligned} & y^{(t)}_{i} =W_{3}(f(W_{2}x_{i}+b_{2})+b_{3}), \\ & p^{(t)}_{i,j} =\log\left(\frac{\exp\left(y^{(t)}_{i,j}\right)}{\sum_{k} \exp\left(y^{(t)}_{i,k}\right)}\right),  \end{aligned}  $$

where *W*_2_ and *W*_3_ are the weight matrices of the two linear layers, respectively, *b*_2_ and *b*_3_ are the bias vectors, and *f*(·) is the activation function. The softmax layer transforms the $y^{(t)}_{i}$ to the trigger label probability vector $p^{(t)}_{i}$. The event trigger label of the *i*-th word is assigned as the *m*-th trigger type where *m* is the index of the maximal element in $p^{(t)}_{i}$ except for $p^{(t)}_{i,none}$ if $p^{(t)}_{i,m}-p^{(t)}_{i,none}>threshold_{t}$; otherwise we assign *none* to the word. The trigger label is then transformed to *t*_*i*_ by the event trigger label embedding layer and then sent to the next time step. Finally, we obtain the sequence of trigger label embedding vectors *T*={*t*_1_,*t*_2_,...,*t*_*n*_}.

Here we define a support value to measure the confidence of the assigned label.

#### **Definition 1**

A **Support Value** is the probability difference between the assigned label *m* and the label *none*. The lager support value means the more confidence of the recognised label.

For each recognized event trigger with index *m*, a support value is computed by:

5 $$ s^{(t)} = p^{(t)}_{m}-p^{(t)}_{none},   $$

where *none* is the index of none type.

In the training of TR, we use a *scheduled sampling* trick to eliminate the gap between training anf inference [33]. We take inverse sigmoid decay function *ε*=*k*/(*k*+ exp(*i*/*k*)) to decide the probability of using true token or inference token, where *i* is the number of training epochs.

### Relation classification

The Relation Classification (RC) module predicts the relation type for each candidate pair. The sentence representation is derived from BiLSTM, and the event trigger is detected by the TR before. Here, we use the information from the events/entities and the sub-sentence between them to predict the type of relation.

Since the TR module has detected the event triggers and the entities are given, we combine all possible trigger-entity and trigger-trigger pairs. The set of candidate pairs is written as *RL*={(*e*^(1)^,*e*^(2)^)|*e*^(1)^∈*event*_*trigger*_*set*,*e*^(2)^∈*event*_*trigger*_*set*∪*entity*_*set*}. To predict the relation type of (*e*^(1)^,*e*^(2)^)∈*RL*, we first take the truncated sequence of BiLSTM hidden vectors $H_{start_{1}:end_{1}}$, the sequence of trigger label vectors $T_{start_{1}:end_{1}}$ and entity label vectors $E_{start_{1}:end_{1}}$, where *start*_1_,*end*_1_ are the start and end location of *e*^(1)^. The three vectors are concatenated into $src = [H_{start_{1}:end_{1}}, T_{start_{1}:end_{1}}, E_{start_{1}:end_{1}}]$. Then, we get $dst = [H_{start_{2}:end_{2}}, T_{start_{2}:end_{2}}, E_{start_{2}:end_{2}}]$ in same manner for *e*^(2)^. The sub-sentence between *e*^(1)^ and *e*^(2)^ is denoted as $mid = [H_{end_{1}:start_{2}}, T_{end_{1}:start_{2}}, E_{end_{1}:start_{2}}]$. Here, we assume *end*_1_<*start*_2_ without loss of generality. If *e*^(1)^ and *e*^(2)^ are adjacent, we assign a learnable vector to *mid* to represent this situation.

The *src* and *dst* are then fed to the adaptive max pooling layer to get a vector with a fixed shape that is denoted as *src*_*max*_ and *dst*_*max*_, which is the same as Eq. 2. Meanwhile, the sub-sentence *mid* is fed to a convolutional layer and an adaptive max pooling layer to extract the feature vector, which is denoted as *mid*_*max*_, same as Eq. 1.

Additionally, Zheng et al. [20] mentioned that the distance of two entities *start*_2_−*end*_1_ can determine the relation significantly. Therefore, we use a distance embedding vector to provide extra information that is ignored by the max pooling operation, and the vector is denoted as *d*_*v*_.

The above vectors are concatenated into *r*=[*src*_*max*_,*mid*_*max*_,*dst*_*max*_,*d*_*v*_] and apply two linear layers:

6 $$ y^{(r)} = W_{5}(f(W_{4}r+b_{4})+b_{5}),   $$

where *W*_4_,*W*_5_ and *b*_4_,*b*_5_ are the weight matrices and bias vectors of two linear layers, respectively. Finally, the vector is fed into a log softmax layer as follows:

7 $$ p^{(r)}_{j} =\log\left(\frac{\exp\left(y^{(r)}_{j}\right)}{\sum_{k} \exp\left(y^{(r)}_{k}\right)}\right),   $$

where $p^{(r)}\in \mathbb {R}^{c}$ is the probability that the relation belongs to each relation class of total *c* classes. We assign the *m*-th class of relation to the candidate pair (*e*^(1)^,*e*^(2)^) where *m* is the index of the maximal element of *p*^(*r*)^ except for $p^{(r)}_{none}$ if $p^{(r)}_{m}-p^{(r)}_{none}>threshold_{r}$; otherwise we assign *none* to the relation.

For each recognized relation with class index *m*, we compute a support value by:

8 $$ s^{(r)} = p^{(r)}_{m}-p^{(r)}_{none},   $$

where *none* is the index of none type.

### Event evaluation

Since the entities, event triggers and relations have been recognized, we then apply the Event Evaluation (EE) module to estimate the probability that a candidate event structure is a valid event.

We use another BiLSTM to represent the event structure. We have obtained the word encoding sequence *H*={*h*_1_,*h*_2_,...,*h*_*n*_} and the entity label embedding sequence *E*={*e*_1_,*e*_2_,...,*e*_*n*_} from BiLSTM, the trigger label embedding sequence *T*={*t*_1_,*t*_2_,...,*t*_*n*_} from TR, and the recognized relation set $RL\_recognized = \left.\left \{\left (e^{(1)}_{i},relation\_type_{i},e^{(2)}_{i}\right)\right |i=1,2,... \right \}$ from RC. Then, we enumerate all the valid combinations of event triggers and relations that have to meet the structure definition of the certain task. All the valid combinations in a sentence form the candidate events set denoted as *C*.

For each candidate event in *C*, a sequence of role label *R*={*r*_1_*r*_2_,...,*r*_*n*_} is leveraged to represent the role of each word in the candidate event. The *i*-th item in *R* is assigned to *event*_*trigger* or *relation*_*type*_*k*_ or *none*_*type* according to the role that the *i*-th word plays. The role label sequence is then transformed into vectors by the role label embedding layer, and the result is denoted as $V^{(e)} = \left \{v^{(e)}_{1},v^{(e)}_{2},...,v^{(e)}_{n}\right \}$.

Then, we concatenate each corresponding vector from sequence *H*, *E*, *T* and *V*^(*e*)^ in parallel, which is written as *X*={*x*_1_,*x*_2_,...,*x*_*n*_} where $x_{i} = [h_{i},e_{i},t_{i},v^{(e)}_{i}]$. The sequence *X* is fed to another BiLSTM layer:

9 $$ h^{(e)}_{i}=BiLSTM(x_{i}), i=1,2,...,n.   $$

The last outputs from two directions of BiLSTM $h^{(e)}_{last}=\left [\overrightarrow {h^{(e)}_{n}},\overleftarrow {h^{(e)}_{1}}\right ]$ are fed to a full connection layer and then mapped to log probability $p^{(e)}\in \mathbb {R}^{2}$ by a log softmax layer. The probability vector *p*^(*e*)^ denotes that the event is a positive sample or a negative sample. Meanwhile, we use another full connection layer and log softmax layer, which is branched from $h^{(e)}_{last}$, to learn a vector $p^{(m)} \in \mathbb {R}^{3}$ that denotes the modification information of the event. The three items in *p*^(*m*)^ represents the log-probability of *Negation*, *Speculation* and *None*, respectively.

For each candidate event, we compute a support value by:

10 $$ s^{(e)} = p^{(e)}_{1}-p^{(e)}_{2},   $$

where $p^{(e)}_{1}$ is the log probability that the candidate event is a valid event and $p^{(e)}_{2}$ is not.

After we obtain the probability vectors *p*^(*t*)^,*p*^(*r*)^,*p*^(*e*)^ and *p*^(*m*)^ from TR, RC and EE, an end-to-end training is then applied to optimize the NLLloss of these vectors according to the correct labels of triggers, relations, events and modifications, respectively.

### Combination strategy

After we have obtained the support value of each candidate trigger, relation and event, the Combination Strategy (CS) is then applied to integrate all of the information and determine the set of final extracted events in prediction phase. CS does not participate in training.

The principle of CS is to minimize the penalty value, which is designed to measure the discordance between the final events and the outputs of previous modules. The penalty consists of two parts as follows. If a trigger or a relation has a positive support value, but it does not appear in any event of the final extracted events, it generates a penalty called “support waste”. In contrast, if a candidate event with a negative support value is included as one of the final extracted events, it generates a penalty called “support lacking”. These two penalties are conflicting objectives. The more candidate events that are added into the final set, the less “support waste” penalty will be, but the penalty for “support lacking” will increase, and vice versa. So, the goal will be to minimize the total penalty so that the final extracted events can be determined.

Formally, this target is written as follows:

11 $$ c_{best}=\mathop{\arg\min}_{c\subseteq C} penalty\_score(c),   $$

where *c*_*best*_ is the set of final extracted events, *c* enumerates all the subsets of *C*, and:

12 $$ \begin{aligned} penalty\_score(c)=\left(\sum_{event_{k}\in c}\max(1-\alpha\cdot s^{(e)}_{k},0)+ \right.\\ \left.\beta\sum_{trigger_{i}\notin c}s^{(t)}_{i}+\gamma\sum_{relation_{j}\notin c}s^{(r)}_{j}\right),  \end{aligned}  $$

where $s^{(e)}_{k}$ denotes the support of the *k*-th candidate event, $s^{(t)}_{i}$ denotes the support of *i*-th event trigger, and $s^{(r)}_{j}$ denotes the support of the *j*-th relation. The first term accounts for the support lacking penalty, the last two terms accounts for the support waste penalty and reweighted by parameters *α*, *β* and *γ*.

**Fig. 4:**
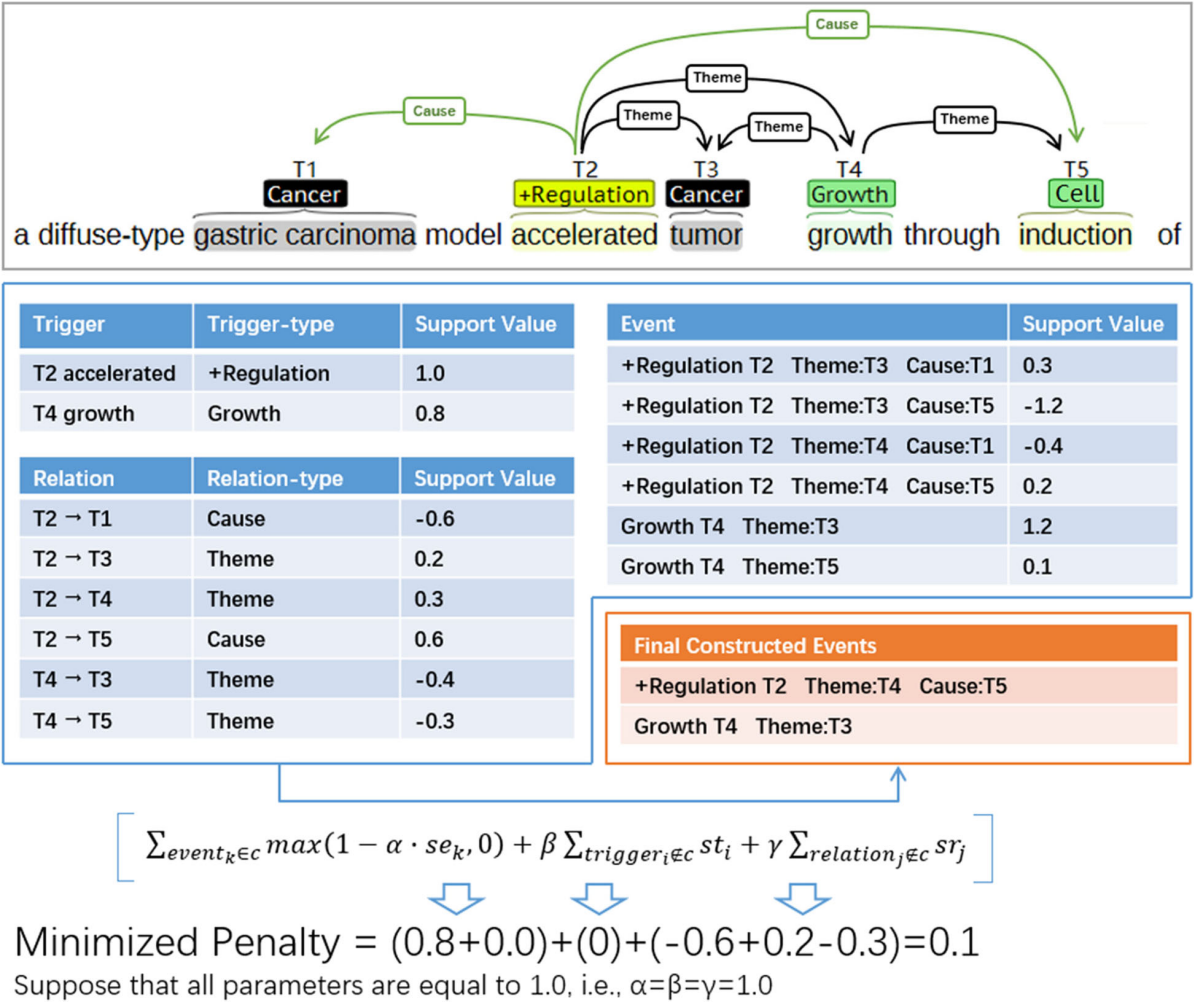
A simple example to illustrate the principle of CS

Figure 4 is a simple example to illustrate the principle of CS. As shown in Fig. 4, three entities (T1, T3 and T5) were given, and two event triggers (T2 and T4) and six relations were detected, then a total of six candidate events were constructed from them. After computing them, the minimized penalty was obtained when choosing the two of them to be the final result.

To optimize Eq. 11, we have to enumerate all of the subsets of *C*, which requires exponential time complexity. For some sentences, there are many complex events with many candidate arguments and lead to a very large computation cost (2^6^ in Fig. 4 for example). Therefore, the CS, which is an approximation algorithm, is proposed to solve the problem within the *O*(*n*^2^) time complexity.

The CS receives the set of candidate events *C*, the support value of triggers, relations and candidate events in a sentence, and then it returns the set of final extracted events. Before the CS, the candidate events are sorted by the topological order of their nested structure if it exist, and the events relating to different triggers are handled independently, except for transmitting the support value between them. In the CS, the initial set of chosen events is empty, and then the candidate events are added into the set one by one in a greedy manner. The pseudo code of the CS is shown in Algorithm 1.

To prevent the nested events from forming event loops, we set a loop detector after CS. The extracted events are added into the final set one-by-one, we discard the event if the inclusion of it would cause event loop. We assign event modifications (sepculation/negation or none-modification) for the events in the final extracted set according to their modification vectors *p*^(*m*)^.


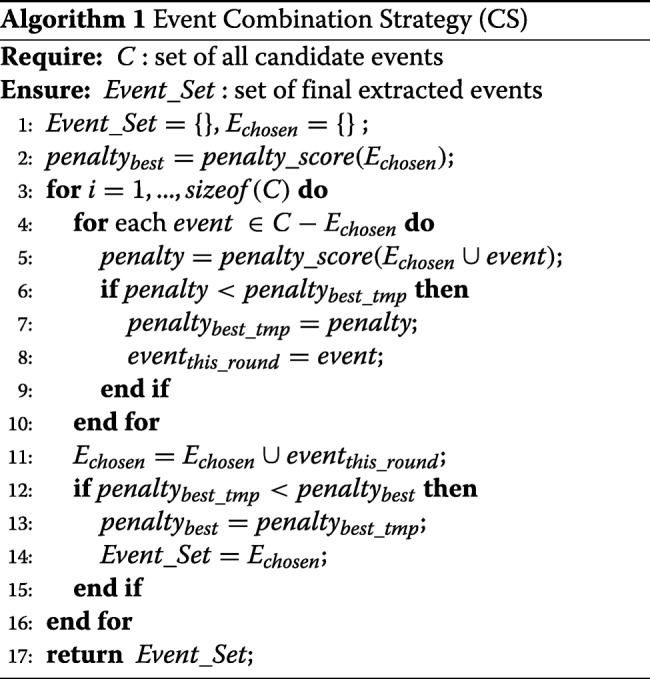


There are two advantages of the CS. First, the CS can address event triggers and relations with negative support value. In the TR and RC, we can set negative thresholds *threshold*_*t*_ and *threshold*_*r*_ to obtain more candidate event triggers and relations, which is especially useful for ensemble learning to improve recall. Second, the CS can alleviate the error accumulation effectivelly. Even though a true event trigger is wrongly assigned a negative support value (TR fails), but the related arguments and event structure are recognized as positive instances (RC and EE work), the trigger can still be correctly constructed as a final event with the CS.

## Supplementary information

**Additional file 1** Supplementary.pdf contains detailed structure of TR/RC/EE (**Figure S1**/**Figure S2**/**Figure S3**), statistics of event structure for CG/PC/MLEE (**Table S1**/**Table S2**/**Table S3**), performance of experiments for CG/PC/MLEE (**Table S4**/**Table S5**/**Table S6**), t-test result for performance of CG/PC/MLEE (**Table S7**/**Table S8**/**Table S9**), statistics of the ignored cases (**Table S10**) and detailed hyper-parameters (**Table S11**).

## Data Availability

The source code is available at https://github.com/melissa135/Event_Extraction. The biomedical event extraction implementation is available online at http://www.predictor.xin/event_extraction. The datasets that used in experiments are available online at http://2013.bionlp-st.org/tasksand http://nactem.ac.uk/MLEE/.

## Abbreviations

CG: Cancer genetics
CNN: Convolutional neural network
CS: Combination strategy
EE: Event evaluation
LR: Linear Regression
LSTM: Long-short term memory
MLEE: Multi-level event extraction
NLP: Natural language processing
PC: Pathway curation
POS: Part of speech
RC: Relation classification
RNN: Recurrent neural network
SGD: Stochastic gradient descent
SVC: Support vector classification
SVM: Support vector machine
TR: Trigger recognition
