# Supplementary material for: Biomedical event extraction with a novel combination strategy based on hybrid deep neural networks
Source: BMC Bioinformatics. 2020 Feb 6;21:47. doi: 10.1186/s12859-020-3376-2 (PMC7006190; doi:10.1186/s12859-020-3376-2)
Supplement: Supplementary file 1 — Additional file 1 Supplementary.pdf contains detailed structure of TR/RC/EE (Figure S1/Figure S2/Figure S3), statistics of event structure for CG/PC/MLEE (Table S1/Table S2/Table S3), performance of experiments for CG/PC/MLEE (Table S4/Table S5/Table S6), t-test result for performance of CG/PC/MLEE (Table S7/Table S8/Table S9), statistics of the ignored cases (Table S10) and detailed hyper-parameters (Table S11). [file 12859_2020_3376_MOESM1_ESM.pdf]

Supplementary Material: Biomedical Event Extraction with a Novel Combination Strategy Based on Hybrid Deep Neural Networks

Lvxing Zhu and Haoran Zheng

December 20, 2019

Contents

Figure 1 Network Structure of Trigger Recognition 3

Figure 2 Network Structure of Relation Classification 4

Figure 3 Network Structure of Event Evaluation 5

Table 1 Structure Statistics for CG 6

Table 2 Structure Statistics for PC 9

Table 3 Structure Statistics for MLEE 11

Table 4 Detailed Performance of CG 13

Table 5 Detailed Performance of PC 14

Table 6 Detailed Performance of MLEE 15

Table 7 $\mathrm{t-test}$ on Performance of CG 16

Table 8 $\mathrm{t-test}$ on Performance of PC 17

Table 9 $\mathrm{t-test}$ on Performance of MLEE 18

Table 10 Statistics for Ignored Cases 19

Table 11 Main $\mathrm{Hyper-parameters}$ of Proposed Model 20

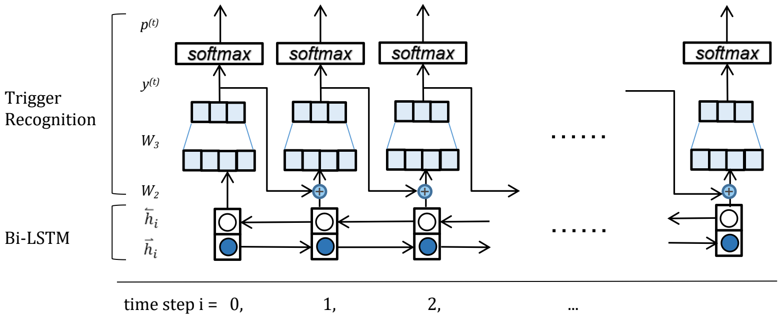

Figure 1: The detail networks structure of Trigger Recognition module.

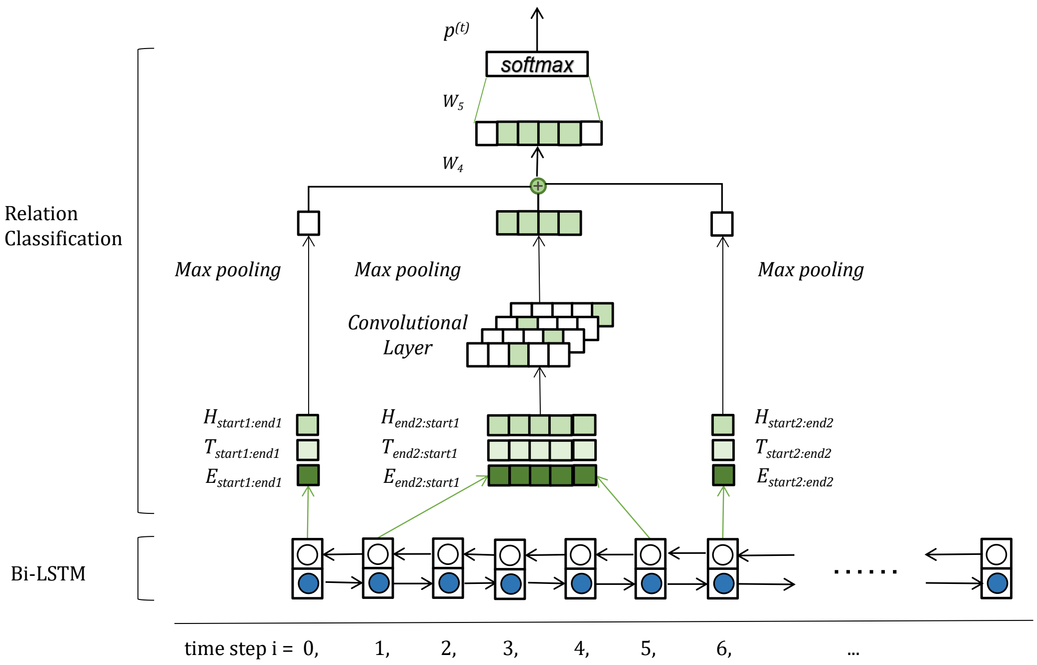

Figure 2: The detail networks structure of Relation Classification module.

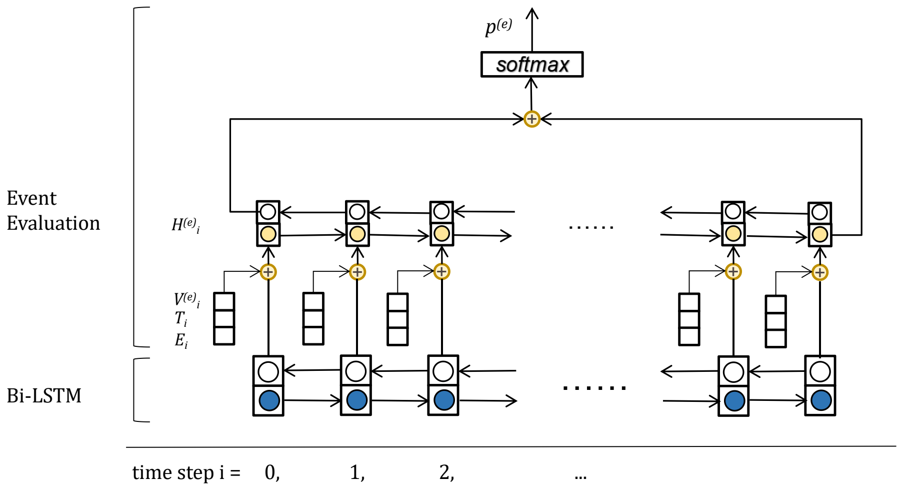

Figure 3: The detail networks structure of Event Evaluation module.

Table 1: The statistics for event structure of BioNLP-ST2013 Cancer Genetic(CG)

Event	Arguments	Count
Acetylation	Theme	5
	Site,Theme	1
Amino_acid_catabolism	-	3
Binding	Site,Theme,Theme2	16
	Theme,Theme2,Theme3	4
	Theme,Theme2	82
	Site,Theme	2
	Theme,Theme2,Theme3,Theme4,Theme5,Theme6	1
	Theme	172
Blood_vessel_development	-	418
	Theme	14
	AtLoc	142
	AtLoc,Theme	2
Breakdown	Theme	94
Carcinogenesis	AtLoc,Theme	2
	Theme	8
	AtLoc	67
	-	93
Catabolism	Theme	36
Cell_death	Theme	93
	-	172
Cell_differentiation	Theme	72
	AtLoc	6
	-	2
Cell_division	Theme	3
Cell_proliferation	Theme	258
Cell_transformation	AtLoc,Theme	2
	-	49
	AtLoc	10
	Theme	140
DNA_demethylation	Theme	1
DNA_methylation	Theme	19
	Site,Theme	17
Death	Theme	140
Dephosphorylation	Theme	4
	Site,Theme	4
Development	Theme	347
Dissociation	Theme,Theme2	1
	Theme	3
Gene_expression	Theme,Theme2,Theme3	4
	Theme,Theme2	11

	Theme,Theme2,Theme3,Theme4,Theme5	1
	Theme	954
Glycolysis	-	49
Glycosylation	Theme	4
Growth	Theme	153
Infection	Theme	10
	Participant,Theme	19
	Participant	5
	-	3
Localization	FromLoc,Theme	30
	FromLoc,Theme,ToLoc	4
	Theme,ToLoc	63
	Theme	433
	AtLoc,Theme,Theme2	1
	Theme,Theme2	5
	AtLoc,Theme	83
Metabolism	Theme	62
Metastasis	Theme,ToLoc	51
	Theme	112
	ToLoc	76
	-	130
Mutation	Theme	86
	-	41
	AtLoc,Theme	49
	Site,Theme	19
	AtLoc,Site,Theme	15
	AtLoc	8
	AtLoc,Theme2	3
	Site	3
Negative_regulation	Cause,Site,Theme	3
	Cause,Theme	901
	Cause,Theme2	1
	CSite,Cause,Theme	9
	Theme	564
Pathway	Participant,Theme	1
	Participant,Participant2,Participant3	7
	Theme	28
	Participant,Participant2	32
	-	37
	Participant,Participant2,Participant3,Participant4	7
	Participant	116
Phosphorylation	Theme	77
	Site,Theme	19
Planned_process	Instrument,Instrument2	5
	Instrument,Instrument2,Theme	20

	Instrument2,Theme	1
	Instrument	216
	Instrument,Instrument2,Instrument3,Theme	3
	Theme	358
	-	3
	Theme,Theme2	11
	Instrument,Theme	355
Positive_regulation	Site,Theme	2
	Cause2,Theme	1
	Theme	984
	CSite,Cause,Theme	6
	CSite,Theme	1
	Cause,Site,Theme	9
	Cause,Theme	1411
Protein_processing	Theme	15
Regulation	CSite,Cause,Theme	8
	Cause,Theme2	1
	Cause2,Theme	1
	Cause,Theme	1042
	Cause,Site,Theme	2
	Theme	261
	Site,Theme	2
Remodeling	Theme	33
Reproduction	Theme	1
Synthesis	Theme	42
Transcription	Theme	113
Translation	Theme	13
Ubiquitination	Theme	4
Total		11718

* The statistics is obtained from training and development dataset of CG.

Table 2: The statistics for event structure of BioNLP-ST2013 Pathway Curation(PC)

Event	Arguments	Count
Acetylation	Site,Theme	10
	Theme	25
	Cause,Site,Theme	4
	Cause,Theme	15
Activation	Theme	316
	Cause,Theme	160
Binding	Theme,Theme2	463
	Theme	299
	Theme,Theme2,Theme3,Theme4	3
	Theme,Theme2,Theme3	27
	Product,Theme	6
	Theme,Theme3	2
	Product	22
Conversion	Product	43
	Product,Theme	49
	Theme	53
	Theme,Theme2	9
	Product,Theme,Theme2	2
Deacetylation	Theme	5
Degradation	Theme	82
Demethylation	Cause,Theme	1
	Site,Theme	1
	Theme	2
Dephosphorylation	Cause,Site,Theme	6
	Cause,Theme	3
	Theme	13
	Site,Theme	4
Deubiquitination	Theme	3
Dissociation	Product,Product2,Product3	1
	Theme	22
	Product	30
	Product,Product2,Product3,Product4	2
	Product,Theme	8
	Product,Product2	9
Gene_expression	Theme	509
Inactivation	Cause,Theme	17
	Theme	82
Localization	FromLoc,Theme,ToLoc	1
	AtLoc,Theme	62
	Theme,ToLoc	29
	Theme	40

Methylation	Cause,Theme	1
	Theme	5
	Cause,Site,Theme	2
	Site,Theme	5
Negative_regulation	Cause,Theme	670
	Theme	418
Pathway	Participant,Participant2	66
	Participant,Participant2,Participant3,Participant4	9
	-	271
	Participant,Participant2,Participant3,Participant4,Participant5	2
	Participant,Participant2,Participant3	12
	Participant	225
Phosphorylation	Theme	262
	Site,Theme	131
	Cause,Theme	119
	Cause,Site,Theme	62
Positive_regulation	Theme	524
	Cause,Theme	1567
Regulation	Cause,Theme	671
	Theme	264
Transcription	Theme	90
Translation	Theme	15
Transport	FromLoc,Theme	28
	Theme,ToLoc	56
	FromLoc,Theme,ToLoc	20
	Theme	141
Ubiquitination	Site,Theme	3
	Cause,Theme	7
	Theme	33
Total		8119

* The statistics is obtained from training and development dataset of PC.

Table 3: The statistics for event structure of MLEE

Event	Arguments	Count
Acetylation	Theme	3
Binding	Theme,Theme2	1
	Theme	121
	Site,Theme,Theme2	8
	Site,Theme	1
Blood_vessel_development	-	434
	AtLoc,Theme	2
	Theme	14
	AtLoc	111
Breakdown	Theme	49
Catabolism	Theme	21
Cell_division	Theme	1
Cell_proliferation	Theme	96
DNA_methylation	Site,Theme	8
	Theme	3
Death	Theme	63
Dephosphorylation	Site,Theme	4
	Theme	1
Development	Theme	226
Dissociation	Theme	1
Gene_expression	Theme	276
Growth	Theme	112
Localization	FromLoc,Theme,ToLoc	1
	Theme,ToLoc	35
	FromLoc,Theme	8
	Theme	254
	AtLoc,Theme	30
Metabolism	Theme	11
Negative_regulation	Cause,Site,Theme	3
	Cause,Theme	400
	Theme	244
	CSite,Cause,Theme	8
Pathway	Participant,Participant2	13
	-	23
	Participant,Participant2,Participant3,Participant4	3
	Participant,Participant2,Participant3	2
	Participant	44
Phosphorylation	Theme	27
	Site,Theme	2
Planned_process	Theme	173
	Instrument,Instrument2	1
	Instrument	129

	Instrument,Instrument2,Theme	2
	Instrument,Theme	162
Positive_regulation	Theme	358
	Theme,Theme2	1
	Cause,Site,Theme	8
	Cause,Theme	565
	Site,Theme	2
	CSite,Cause,Theme	1
Protein_processing	Theme	6
Regulation	CSite,Cause,Theme	4
	Cause,Theme	422
	Cause,Site,Theme	2
	Theme	110
	Site,Theme	1
Remodeling	Theme	25
Synthesis	Theme	13
Transcription	Theme	21
Translation	Theme	2
Ubiquitination	Theme	1
Total		4673

* The statistics is obtained from training and development dataset of MLEE.

Table 4: Full detailed performance of BioNLP-ST2013 Cancer Genetic(CG)

Event Class	gold (match)	answer (match)	recall	prec.	fscore
Development	208 (161)	213 (161)	77.40	75.59	76.48
Blood_vessel_developm	313 (266)	290 (266)	84.98	91.72	88.23
Growth	72 (62)	67 (62)	86.11	92.54	89.21
Death	59 (49)	66 (49)	83.05	74.24	78.40
Cell_death	121 (92)	118 (92)	76.03	77.97	76.99
Cell_proliferation	126 (86)	98 (86)	68.25	87.76	76.79
Cell_division	1 (0)	2 (0)	0.00	0.00	0.00
Cell_differentiation	37 (18)	41 (18)	48.65	43.90	46.15
Remodeling	15 (6)	8 (6)	40.00	75.00	52.17
Reproduction	0 (0)	0 (0)	100.00	100.00	100.00
=[ANATOMY-TOTAL]=	952 (740)	903 (740)	77.73	81.95	79.78
Mutation	127 (35)	79 (35)	27.56	44.30	33.98
Carcinogenesis	71 (49)	60 (49)	69.01	81.67	74.81
Cell_transformation	92 (74)	88 (74)	80.43	84.09	82.22
Breakdown	49 (37)	42 (37)	75.51	88.10	81.32
Metastasis	169 (130)	167 (130)	76.92	77.84	77.38
Infection	14 (6)	9 (6)	42.86	66.67	52.17
=[PATHOL-TOTAL]=	522 (331)	445 (331)	63.41	74.38	68.46
Metabolism	14 (13)	25 (13)	92.86	52.00	66.67
Synthesis	22 (15)	20 (15)	68.18	75.00	71.43
Catabolism	15 (7)	11 (7)	46.67	63.64	53.85
Glycolysis	22 (22)	23 (22)	100.00	95.65	97.78
Amino_acid_catabolism	4 (2)	2 (2)	50.00	100.00	66.67
Gene_expression	524 (396)	488 (394)	75.57	80.74	78.07
Transcription	55 (33)	39 (33)	60.00	84.62	70.21
Translation	8 (0)	5 (0)	0.00	0.00	0.00
Protein_processing	0 (0)	0 (0)	100.00	100.00	100.00
Acetylation	2 (2)	2 (2)	100.00	100.00	100.00
Glycosylation	0 (0)	0 (0)	100.00	100.00	100.00
Phosphorylation	29 (13)	21 (13)	44.83	61.90	52.00
Ubiquitination	0 (0)	1 (0)	0.00	0.00	0.00
Dephosphorylation	3 (1)	1 (1)	33.33	100.00	50.00
DNA_methylation	24 (9)	17 (9)	37.50	52.94	43.90
DNA_demethylation	2 (0)	0 (0)	0.00	0.00	0.00
Pathway	127 (82)	118 (82)	64.57	69.49	66.94
=[MOLECUL-TOTAL]=	851 (595)	773 (593)	69.92	76.71	73.16
Binding	110 (53)	72 (53)	48.18	73.61	58.24
Dissociation	3 (0)	0 (0)	0.00	0.00	0.00
Localization	285 (153)	223 (153)	53.68	68.61	60.24
=[GENERAL-TOTAL]=	398 (206)	295 (206)	51.76	69.83	59.45
Regulation	637 (214)	498 (214)	33.59	42.97	37.71
Positive_regulation	1076 (394)	630 (390)	36.62	61.90	46.02
Negative_regulation	657 (280)	501 (279)	42.62	55.69	48.28
=[REG-TOTAL]=	2370 (888)	1629 (883)	37.47	54.21	44.31
Planned_process	437 (202)	402 (202)	46.22	50.25	48.15
===[SUB-TOTAL]===	5530 (2962)	4447 (2955)	53.56	66.45	59.31
Negation	259 (95)	163 (94)	36.68	57.67	44.84
Speculation	183 (43)	87 (42)	23.50	48.28	31.61
==[MOD-TOTAL]==	442 (138)	250 (136)	31.22	54.40	39.67
====[TOTAL]====	5972 (3100)	4697 (3091)	51.91	65.81	58.04

* The performance is evaluated by BioNLP official online evaluation.

Table 5: Full detailed performance of BioNLP-ST2013 Pathway Curation (PC)

Event Class	gold (match)	answer (match)	recall	prec.	fscore
Conversion	67 (23)	47 (23)	34.33	48.94	40.35
Phosphorylation	309 (183)	257 (183)	59.22	71.21	64.66
Dephosphorylation	20 (13)	16 (13)	65.00	81.25	72.22
Acetylation	23 (16)	18 (16)	69.57	88.89	78.05
Deacetylation	3 (3)	5 (3)	100.00	60.00	75.00
Methylation	7 (4)	5 (4)	57.14	80.00	66.67
Demethylation	0 (0)	0 (0)	100.00	100.00	100.00
Ubiquitination	17 (8)	14 (8)	47.06	57.14	51.61
Deubiquitination	0 (0)	0 (0)	100.00	100.00	100.00
Localization	71 (27)	43 (27)	38.03	62.79	47.37
Transport	145 (73)	108 (73)	50.34	67.59	57.71
Gene_expression	246 (230)	258 (229)	93.50	88.76	91.07
Transcription	52 (33)	49 (33)	63.46	67.35	65.35
Translation	2 (1)	1 (1)	50.00	100.00	66.67
=[SIMPLE-TOTAL]=	962 (614)	821 (613)	63.83	74.67	68.82
Degradation	42 (34)	49 (34)	80.95	69.39	74.73
Activation	247 (193)	242 (193)	78.14	79.75	78.94
Inactivation	65 (32)	64 (32)	49.23	50.00	49.61
Binding	391 (205)	369 (205)	52.43	55.56	53.95
Dissociation	39 (8)	26 (8)	20.51	30.77	24.62
Pathway	265 (229)	266 (229)	86.42	86.09	86.25
==[NONREG-TOTAL]==	2011 (1315)	1837 (1314)	65.39	71.53	68.32
Regulation	444 (175)	383 (174)	39.41	45.43	42.21
Positive_regulation	1020 (354)	674 (350)	34.71	51.93	41.61
Negative_regulation	529 (222)	417 (219)	41.97	52.52	46.65
==[REG-TOTAL]==	1993 (751)	1474 (743)	37.68	50.41	43.13
===[SUB-TOTAL]===	4004 (2066)	3311 (2057)	51.60	62.13	56.38
Negation	155 (50)	82 (45)	32.26	54.88	40.63
Speculation	19 (0)	0 (0)	0.00	0.00	0.00
==[MOD-TOTAL]==	174 (50)	82 (45)	28.74	54.88	37.72
====[TOTAL]====	4178 (2116)	3393 (2102)	50.65	61.95	55.73

* The performance is evaluated by BioNLP official online evaluation.

Table 6: Full detailed performance of Multi-Level Event Extraction corpus (MLEE)

Event Class	gold (match)	answer (match)	recall	prec.	fscore
Development	107 (88)	117 (88)	82.24	75.21	78.57
Blood_vessel_developm	310 (268)	288 (268)	86.45	93.06	89.63
Growth	60 (48)	53 (48)	80.00	90.57	84.96
Death	38 (26)	41 (26)	68.42	63.41	65.82
Breakdown	24 (15)	18 (15)	62.50	83.33	71.43
Cell_proliferation	45 (30)	36 (30)	66.67	83.33	74.07
Remodeling	10 (3)	3 (3)	30.00	100.00	46.15
=[ANATOMY-TOTAL]=	594 (478)	556 (478)	80.47	85.97	83.13
Synthesis	4 (3)	4 (3)	75.00	75.00	75.00
Catabolism	5 (1)	3 (1)	20.00	33.33	25.00
Gene_expression	164 (129)	168 (129)	78.66	76.79	77.71
Transcription	16 (1)	1 (1)	6.25	100.00	11.76
Phosphorylation	4 (2)	4 (2)	50.00	50.00	50.00
Dephosphorylation	1 (1)	1 (1)	100.00	100.00	100.00
=[MOLECUL-TOTAL]=	194 (137)	181 (137)	70.62	75.69	73.07
Binding	63 (25)	49 (25)	39.68	51.02	44.64
Localization	136 (98)	137 (98)	72.06	71.53	71.79
Regulation	243 (72)	159 (72)	29.63	45.28	35.82
Positive_regulation	404 (168)	318 (168)	41.58	52.83	46.54
Negative_regulation	291 (118)	221 (118)	40.55	53.39	46.09
=[GENERAL-TOTAL]=	1137 (481)	884 (481)	42.30	54.41	47.60
Planned_process	196 (71)	145 (71)	36.22	48.97	41.64
====[TOTAL]====	2121 (1167)	1766 (1167)	55.02	66.08	60.05

* The performance is evaluated on the testset of the MLEE.

Table 7: t-test on performance of baseline method and performance of our proposed method, Cancer Genetics (CG)

	Baseline Performance	Proposed Performance
Mean	57.60%	57.97%
Median	57.60%	58.04%
Variance	-	5.729×10^{-6}
Observations	1	11
t Stat	-5.176	
P($T \leq t$) one-tail	0.0002	

* The baseline performance is the highest F1 score obtained by existing methods.
* We trained 10 single models independently, then randomly chose 5 of them for ensemble learning and repeated for 11 times. The performance of each ensemble learning is 58.32%, 57.45%, 58.08%, 57.91%, 57.86%, 58.09%, 58.04%, 57.95%, 58.16%, 57.71% and 58.14%, respectively.

Table 8: t-test on performance of baseline method and performance of our proposed method, Pathway Curation (PC)

	Baseline Performance	Proposed Performance
Mean	55.62%	55.70%
Median	55.62%	55.73%
Variance	-	2.787×10^{-6}
Observations	1	11
t Stat	-1.680	
$P(T \leq t)$ one-tail	0.0619	

* The baseline performance is the highest F1 score obtained by existing methods.
* We trained 10 single models independently, then randomly chose 5 of them for ensemble learning and repeated for 11 times. The performance of each ensemble learning is 55.73%, 55.78%, 55.56%, 55.51%, 55.39%, 55.8%, 55.82%, 55.66%, 55.93%, 55.9% and 55.67%, respectively.

Table 9: t-test on performance of baseline method and performance of our proposed method, Multi-Level Event Extraction corpus (MLEE)

	Baseline Performance	Proposed Performance
Mean	59.65%	60.04%
Median	59.65%	60.05%
Variance	-	9.937×10^{-6}
Observations	1	11
t Stat	-4.170	
P($T \leq t$) one-tail	0.0009	

* The baseline performance is the highest F1 score obtained by existing methods.
* We trained 10 single models independently, then randomly chose 5 of them for ensemble learning and repeated for 11 times. The performance of each ensemble learning is 60.41%, 59.88%, 60.48%, 59.65%, 60.22%, 60.05%, 60.3%, 60.28%, 59.96%, 59.67% and 59.61%, respectively.

Table 10: Statistics for the ignored cases of CG/PC/MLEE

Corpus	Ratio of SPAN	Ratio of MULTI
CG	2.13%	1.53%
PC	2.73%	1.67%
MLEE	1.58%	0.73%

* The statistics are derived from training set for CG/PC/MLEE.
* *Ratio of SPAN* is the ratio of the events that span across more than one sentences to total events. *Ratio of MULTI* is the ratio of the event triggers that associate with multiple labels to total triggers.

Table 11: Main hyper-parameters of proposed model

Module	Parameter	Value
Char-CNN	size of char embedding (d)	32
	kernel size of Char-CNN (k)	5
	number of output channels (nc)	64
BiLSTM	size of word embedding	192
	size of entity embedding	16
	size of BiLSTM hidden vector (hd)	256
Tigger Recoginition	size of event embedding	16
	dropout rate of inputs	0.2
Relation Classification	size of distance embedding	8
	kernel size of CNN	3
	number of CNN output channels	128
	dropout rate of dense layer	0.2
Event Evaluation	size of role embedding	8
	size of BiLSTM hidden vector	128
